# The Diagnosis and Blistering Mechanisms of Mucous Membrane Pemphigoid

**DOI:** 10.3389/fimmu.2019.00034

**Published:** 2019-01-24

**Authors:** Mayumi Kamaguchi, Hiroaki Iwata

**Affiliations:** ^1^Department of Dermatology, Hokkaido University Graduate School of Medicine, Sapporo, Japan; ^2^Department of Oral Diagnosis and Medicine, Hokkaido University Graduate School of Dental Medicine, Sapporo, Japan

**Keywords:** mucous membrane pemphigoid, type XVII collagen, direct immunofluorescence, collagen IV, C-terminas, steric hindrance

## Abstract

Mucous membrane pemphigoid (MMP) is a mucous membrane-dominated autoimmune subepithelial blistering disease that is caused by autoantibodies against various autoantigens in basement membrane zone (BMZ) proteins, including collagen XVII (COL17). Clinicians face diagnostic problems in detecting circulating antibodies and targeted antigens in MMP. The diagnostic difficulties are mainly attributed to the low titers of MMP autoantibodies in sera and to heterogeneous autoantigens. Additionally, no unanimous diagnostic criteria have been drawn for MMP, which can result in delayed diagnoses or misdiagnoses. This review aims to integrate and present currently available data to clarify diagnostic strategies and to present diagnostic criteria for MMP. The ultimate blistering mechanism in MMP has not been elucidated, and such mechanism is especially obscure in COL17-type MMP. In bullous pemphigoid (BP), which is the most common autoimmune subepidermal blistering disease, some patients show oral lesion as well as predominant skin lesions. However, there is no fundamental explanation for the onset of oral lesions in BP. This article summarizes innovative research perspectives on the pathogenesis of oral lesions in pemphigoid. Finally, we propose a potential pathogenesis for COL17-type MMP.

## Introduction

Mucous membrane pemphigoid (MMP) refers to mucous membrane-dominated autoimmune subepithelial blistering diseases (1–4). MMP is caused by autoantibodies against various autoantigens in the basement membrane zone (BMZ), including collagen XVII (COL17, also called BP180) (5), BP230 (6), laminin 332 (7–9), integrin α6/β4 (10–12), and collagen VII (COL7) (13, 14). Of these, the C-terminus of COL17 and laminin 332 are thought to be major autoantigens for MMP. Autoantibodies against Integrinα 6/β4 are associated with the occurrence of ocular lesions (15). Clinically, the most common site of involvement in MMP is the oral mucosa (80–90%). Also involved are the ocular mucosa (50%), the skin (20%), the genital mucosa (15%), the anal mucosa (10%), and the pharynx, esophagus and larynx (<10%) (16). In the oral cavity, the gingiva is most commonly affected (70% of oral MMP cases), followed by the buccal mucosa (60%), the palate (27%), and the tongue and lips (13%) (17).

MMP is relatively difficult to diagnose clinically, for several reasons that are documented in the next section. One reason is that the titer of autoantibodies is lower in MMP than in bullous pemphigoid (BP) (18, 19), which is the most common autoimmune skin blistering disease (2). Therefore, circulating autoantibodies are detected less frequently in MMP than in BP (17), which leads to challenges in diagnosing MMP.

The pathogenesis of MMP has been poorly understood to date. As for the mechanism of blister formation in autoimmune blistering disorders, the direct inhibition of protein–protein binding by autoantibodies in pemphigus (steric hindrance) (20, 21) and/or Fc-mediated complement and inflammatory cell activation in pemphigoid, including in epidermolysis bullosa acquisita, have been reported (22, 23). However, the mechanism of blister formation on the oral mucosa in pemphigoid remains undiscovered. Histologically, MMP patients tend to have fewer inflammatory findings than BP patients do (24). This may indicate differences in blistering mechanisms between MMP and BP.

This article focuses on diagnostic tips for improving the clinical diagnosis of MMP and proposes a possible pathomechanism for oral mucosa-specific blister formation that is related to less severe inflammatory mechanisms.

## Issues in Diagnosing MMP

To diagnose MMP, we perform several tests, including histological and immunological analyses. Histologically, the formation of junctional separations at the BMZ is observed in specimens from lesional mucosa (25, 26). However, histopathological examination is not always performed, particularly when the lesions are limited to the eyes due to the hesitation about performing conjunctival biopsies and the concern about scar formation. In addition, such examination does not always reveal subepithelial blisters, because of tissue destruction (27). Immunologically, the deposition of IgG autoantibodies and C3, or sometimes of IgA autoantibodies, at the BMZ can be detected by direct immunofluorescence (DIF) of lesional or perilesional samples (1, 28). To identify circulating autoantibodies to the BMZ, indirect immunofluorescence (IIF) with normal human skin as the substrate is usually performed, but autoantibodies are detected in only 17–53% of MMP cases (5, 17, 18, 29). According to the latest data from our hospital, in 22% (8/36) of cases, MMP autoantibodies (IgG) are detected by IIF with normal human skin. 1 M NaCl-split skin IIF (ssIIF) is more sensitive than IIF. The staining of ssIIF with MMP sera produces linear IgG deposits in 58–82% of MMP cases on either the epidermal or the dermal side of the BMZ (17, 30, 31). Even though ssIIF might be helpful to differentiate autoantigens located on the epidermal side of the BMZ from those located on the dermal side of the BMZ by causing separation at the level of the lamina lucida, it does not definitively identify the autoantigens. Immunoblotting using epidermal or dermal extracts and recombinant antigenic polypeptides is useful for identifying specific targeted autoantigens, although the techniques are not commonly available in routine examinations. Enzyme-linked immunosorbent assays (ELISAs) are widely used to detect autoantibodies directing specific autoantigens (32, 33). Approximately 85–96% of autoantibodies in BP are detected by commercially available COL17-NC16A ELISA or chemiluminescence enzyme immunoassay (CLEIA) systems (33–36). However, the antigens targeted by commercially available ELISA/CLEIA systems are limited to the certain domains on BMZ proteins, such as COL17 (NC16A), BP230 (N/C-terminus), and COL7 (NC1/NC2) (37). Due to the low titer and the heterogeneity of MMP autoantibodies, only 30–52% of the autoantibodies in COL17-type MMP may be recognized using COL17 NC16A ELISA/CLEIA (17, 38, 39). Because of these problems, a certain share of MMP patients may not be diagnosed and treated.

### Diagnostic Points for MMP

#### Structure-Maintaining Biopsy Technique for Hematoxylin and Eosin (H&E) Staining

Inadequate biopsy techniques and improper tissue handing can easily lead to the loss of the oral mucosal epithelia in samples from MMP patients. The destruction of oral mucosal tissue makes a diagnosis difficult. Endo et al. presented a “stab-and-roll” biopsy technique to maintain the gingival epithelia in desquamative gingivitis (40). This technique is designed to keep the epithelium from detaching from the biopsy specimen by reducing lateral forces during the procedure. In 52 patients with desquamative gingivitis, the epithelium was maintained in 12 of the 13 patients with MMP using this technique.

#### Tissue-Bound Immunoglobulin and Complement

In cases that are difficult to diagnose, DIF using the patient's tissue is a valuable test for diagnosing MMP. Shimanovich et al. showed that multiple and repeated biopsies increase the sensitivity of DIF (41). At the first workup, 69% (36/52) of patients who underwent only 1 biopsy were found to be positive, whereas 85% (22/26) of patients who underwent biopsies from more than 1 site were positive. Overall, 95% (74/78) showed positive results in DIF after repeated biopsies. The same group demonstrated that immunohistochemistry for C3d or C4d is helpful in screening for cases of suspected MMP when paraffin-embedded tissue is available (42). Linear deposits of C3d or C4d were detected in 53% (18/34) or 59% (20/34) of patients, respectively. We also reported that DIF samples taken from non-lesional buccal mucosa by punch biopsy contribute to the diagnosis of MMP (27). In 7 MMP with gingiva-dominant oral lesions, tissue-bound antibodies were detected in all cases. The buccal mucosa is easy to access, and punch biopsies provide well-maintained BMZ structures.

#### Detection of Autoantibodies

##### Laminin 332-Type MMP

Goletz et al. established a specific IIF assay using laminin 332-expressing human HEK293 cells for the detection of anti-laminin 332 autoantibodies (43). Using BIOCHIP® mosaics, the laminin 332 heterotrimer recognized 77% (72/93) of the anti-laminin 332 MMP sera. Several studies developed ELISA systems to detect autoantibodies against laminin 332. Bernard et al. presented a novel ELISA that uses purified laminin 332 from SCC25 cells and detected autoantibodies in 20.1% of MMP patients (44). Chiorean et al. showed that the results of an ELISA using purified or recombinant human laminin 332 correlated closely with those of immunoblotting in 36 MMP cases (45).

##### COL17-Type MMP

Ali et al. demonstrated that salivary IgG and IgA antibodies against COL17-NC16A are equal to serum in diagnostic value (39). 45% (29/64) of whole saliva samples and 53% (33/64) of serum samples were positive for IgG and/or IgA antibodies by COL17-NC16A ELISA. However, 50–70% of MMP autoantibodies were found to mainly react with the C-terminus of COL17 instead of with the COL17-NC16A domain (5, 6, 46). Immunoassays for the detection of autoantibodies against the entire extra domain of COL17 need to be provided. Schmidt et al. proposed an IF assay that uses Sf21 insect cells expressing full-length COL17 (47). The novel assay detected 84% (6/7) of circulating autoantibodies in MMP sera. Recently Izumi et al. established a novel ELISA system using full-length human COL17 recombinant protein (full-length COL17 ELISA) (48). With the full-length COL17 ELISA, 9 of 12 MMP cases (75%) showed positivity, whereas with the conventional COL17-NC16A ELISA and BP230 ELISA, 4 of 12 sera (42%), and 3 of 12 sera (25%) showed positivity, respectively. We reported on another unique concept that helped to overcome the difficulty of detecting MMP autoantibodies. IIF is usually performed using normal human skin, even though MMP mainly involves the mucosa and not the skin. We performed IIF tests using normal human oral mucosa (18). In 20 MMP sera and 20 BP sera, the sensitivities were compared by IIF using skin and mucosa. 85% (17/20) of the MMP sera reacted to mucosa, and 35% (7/20) to skin. Immunoblotting using normal human epidermal keratinocytes (skin keratinocytes) and normal human oral mucosal keratinocyte lysates was able to detect autoantibodies. Skin and mucosal keratinocyte lysates reacted to a 180-kDa protein corresponding to COL17 in 10% (2/20) and 55% (11/20) of MMP sera, respectively.

Mucosal substrates (normal oral mucosa and normal oral mucosal keratinocytes) are beneficial for detecting autoantibodies and identifying autoantigens in MMP. However, it is more difficult to obtain oral mucosa than skin. To overcome this drawback, we attempted to immortalize the oral mucosal keratinocytes by using E6/E7 proteins of HPV (49). Cell lysates of immortalized mucosal keratinocytes effectively identified MMP autoantigens in 60% (18/30) of MMP sera.

For the sensitive detection of COL17-NC16A-specific IgG, Emtenani et al. reported that normal human skin was superior to monkey esophagus. The monkey esophagus was able to detect only 17% (2/12) of COL17-NC16A antibodies, whereas skin detected 100% (12/12) of the antibodies (50). Together with the low homology of COL17-NC16A between human and monkey esophagus, these pieces of evidence suggest that the expression of COL17 protein differs by anatomical location, such as skin vs. mucosa. The use of suitable specimens for IIF from specific sites may contribute to the sensitive detection of autoantibodies in pemphigoid.

### Proposed Diagnostic Strategies

**Figure 1:**
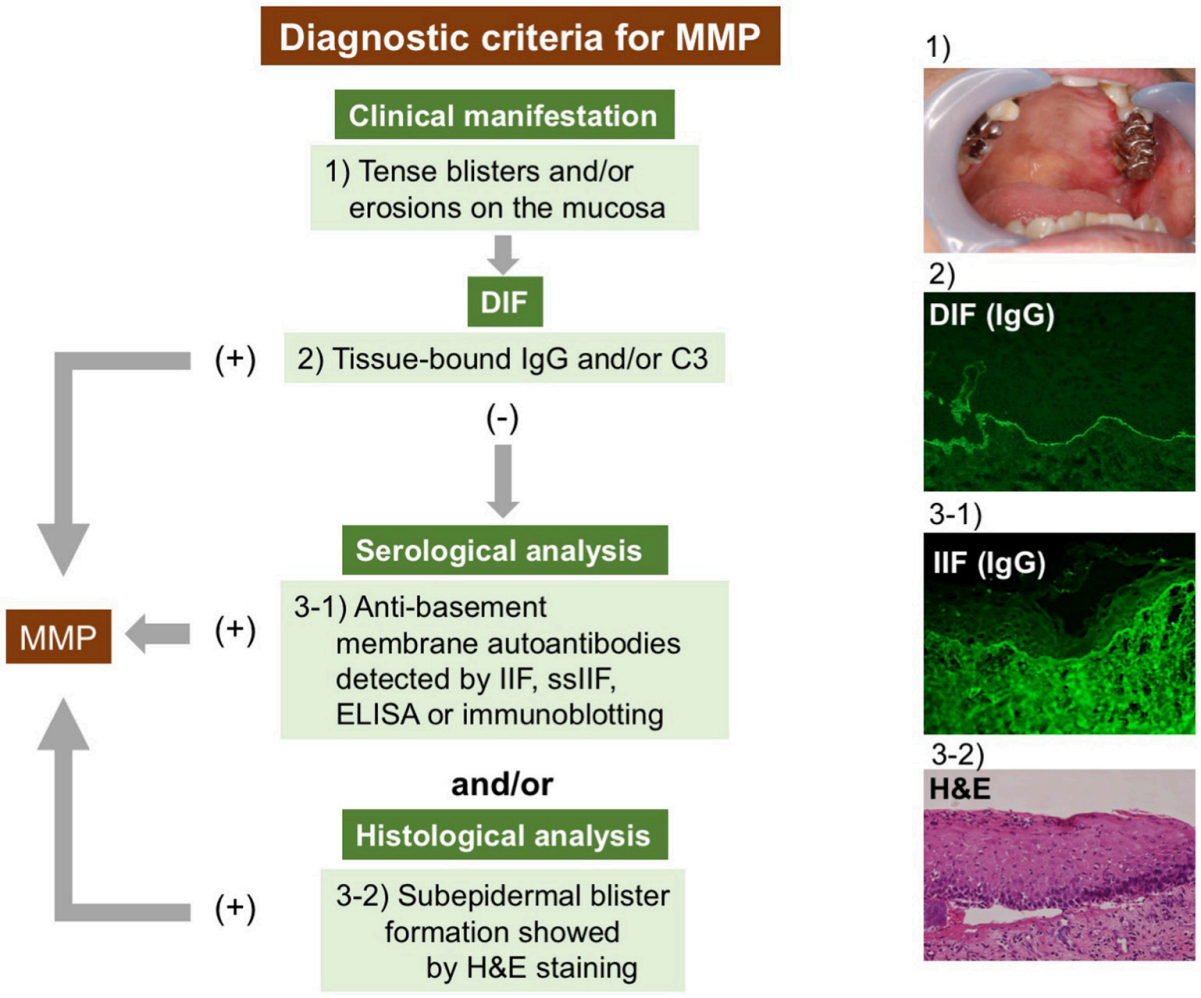
Diagnostic strategy for MMP. The diagnosis of MMP is confirmed by clinical features and positive DIF results. In DIF-negative or DIF-unavailable cases, at least one serological or histological finding is needed. DIF, direct immunofluorescence; IIF, indirect immunofluorescence; ELISA, enzyme-linked immunosorbent assay; H&E, hematoxylin and eosin staining.

The diagnostic criteria for MMP remain unclear. The lack of commonly recognized diagnostic criteria can result in delayed diagnoses or misdiagnoses. The international consensus has documented that the clinical findings of mucosa-dominant lesions and DIF detecting tissue-bound IgG, IgA, and/or C3 are essential for the diagnosis (1). Several studies have followed this criteria (41, 44, 51). We also fundamentally agree with the criteria and have introduced it in our studies. However, in pure ocular pemphigoid, up to 20% of cases are negative in DIF (52, 53). Therefore, serological analyses detecting circulating autoantibodies and histological examinations are included in the diagnostic criteria in some studies (39, 52, 54, 55). To clarify MMP diagnosis, we here propose diagnostic criteria based on current knowledge. We emphasize the significance of serological analyses, especially in cases of DIF negativity (Figure 1). The diagnosis of MMP is confirmed when clinical criteria and DIF findings are fulfilled. In DIF-negative or DIF-unavailable (not performed) cases, at least one serological or histological finding is needed.

## The Evidence of Blistering Mechanisms for Oral Lesions in Pemphigoid

### Clinical Evidence of Mucosal Lesions in Pemphigoid

Several pieces of evidence demonstrate associations between mucosal lesions and certain clinical features in pemphigoid. Kridin et al. reported that BP patients with normal eosinophil counts present mucous lesions more frequently than those with elevated eosinophil counts (56). In some patients with oral involvement, the administration of a dipeptidyl peptidase-4 inhibitor (DPP-4i) is associated with BP onset (57). An association between MMP and the intake of DDP-4i was also reported (58). Furthermore, a certain HLA allele (HLA-DQB1^*^03:01) is associated with MMP occurrence (59–63). HLA-DQB1^*^03:01 is also related to a high risk of DDP-4i-associated BP (59). Hofmann et al. demonstrated that 56% of BP patients with mucosal involvement showed IgG reactivity against both the COL17-NC16A and C-terminus regions of COL17 (64). Clape et al. revealed that the absence of anti-BP230 autoantibodies was associated with the presence of mucosal lesions in BP (65).

### Experimental Evidence of MMP *in vivo* and *in vitro*

In addition to the previous studies on mechanisms of blister formation in BP, several lines of evidence that partly explain the MMP pathogenesis have been demonstrated.

#### Laminin 332-Type MMP

In 2017, Heppe et al. established a mouse model in which injected rabbit anti-laminin 332 IgG antibodies caused subepidermal blisters to develop in the ears, eyes and oral cavity of adult C57BL/6 mice (66). In this model, the clinical manifestations are Fc receptor-dependent and complement-dependent, similar to those of previous BP mouse models. Meanwhile, Lazarova et al. showed that injections with rabbit anti-human laminin 332 IgG or Fab fragments induced subepithelial blisters in the skin of neonatal BALB/c mice (67, 68). In this antibody-transfer model, C5-deficient or mast cell-deficient mice also exhibited subepidermal blisters on the skin (67). The same group established a human skin graft model that uses SCID mice (69). They noted that the injection of purified IgG from MMP patients or anti-human laminin 332 IgG resulted in non-inflammatory subepidermal blisters on the skin. These models from the latter group might define the pathomechanisms in MMP; however, the models lack predominant mucosal involvement.

#### COL17-Type MMP

Although experimental mouse models for laminin 332-type MMP have been established, *in vivo* studies on COL17-type MMP have not made progress. In previous studies of COL17-targeted mouse models for BP, oral lesions were not addressed and there was a lack of description, both clinically and histologically (70–73).

For the pathogenesis of COL17-type MMP *in vitro*, Imanishi et al. reported that some MMP IgGs targeting the C-terminus of COL17 showed the internalization of COL17 in both oral keratinocytes and DJM-1 cells (74), which are cells from a squamous cell carcinoma cell line (75). In contrast, other MMP IgGs against the C-terminus did not induce the internalization of COL17 thoroughly. The autoantibody-induced endocytosis of COL17 is thought to play an important role in pemphigoid blister formation (76, 77). However, it remains unclear why some MMP IgGs targeting the C-terminus of COL17 show the internalization of COL17.

## Potential Blistering Mechanisms in the Oral Mucosa in Pemphigoid

Even though COL17 is a major targeted antigen both in BP and MMP, the predominantly involved organs differ between these two diseases. The blister mechanisms in autoimmune subepidermal blistering diseases are complicated, but they are strongly associated with various factors, such as complement activation and inflammatory cell infiltrates (78, 79). In contrast, several lines of evidence have been reported regarding complement-independent blister formation in BP (80). Anti-COL17-NC16A antibodies induce the internalization and depletion of COL17 and lead to inadequate adhesion strength in keratinocytes (76). COL17 depletion is important for blistering along the lamina lucida without inflammation. We noticed that no research has addressed the pathomechanisms of oral lesions in both BP and MMP. Before addressing the possible pathogenesis of MMP, we first focus on blister formation in the oral mucosa in BP.

### The Blistering Mechanism in the Oral Mucosa in BP

**Figure 2:**
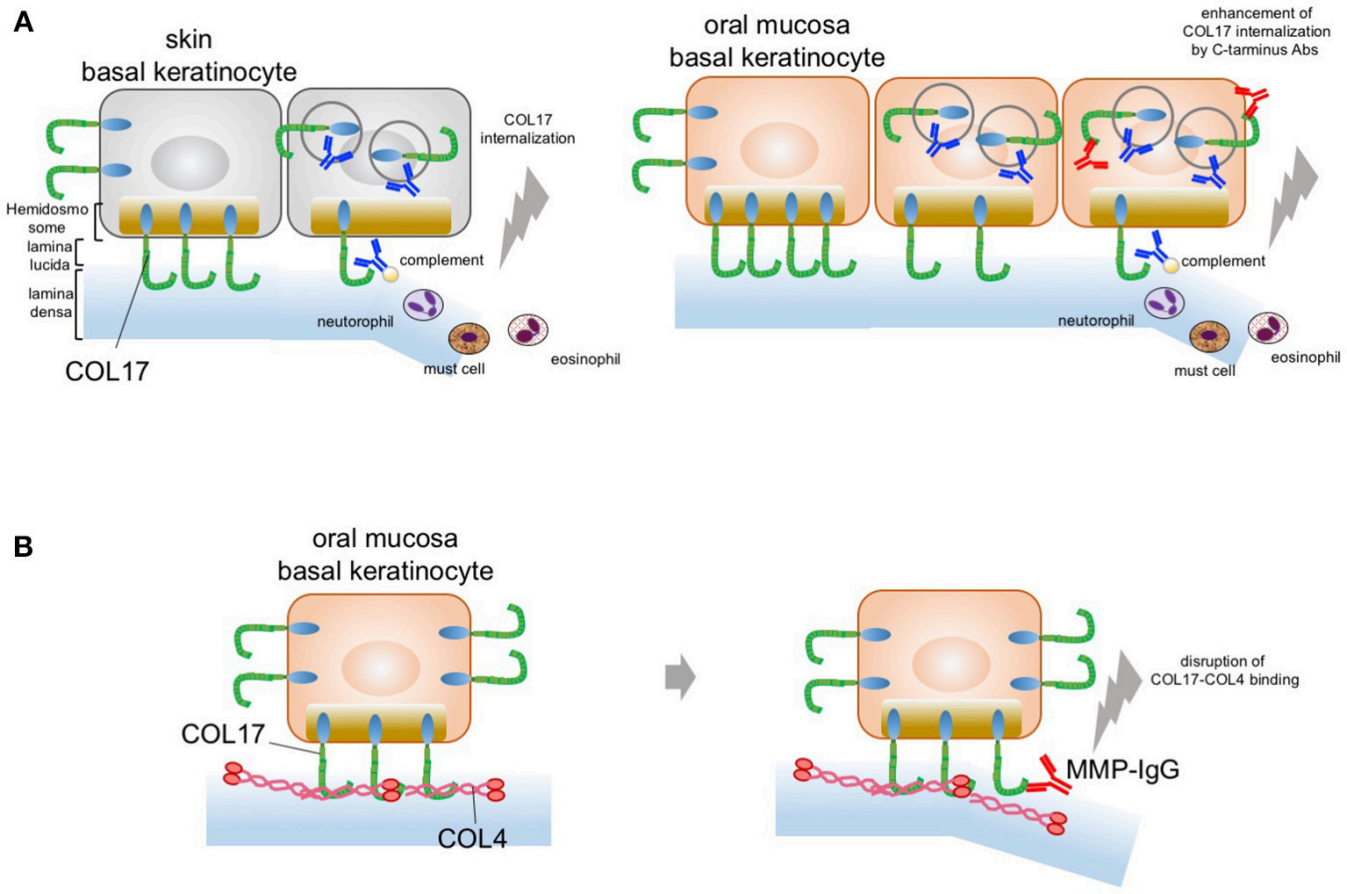
Potential blistering mechanisms in oral mucosa. **(A)** The oral mucosal blistering in BP. COL17 molecules are located in both the hemidesmosomal and the non-hemidesmosomal plasma membranes. In the skin, autoantibodies targeting COL17-NC16A lead to the internalization of non-hemidesmosomal COL17 and result in COL17 depletion. The internalization and depletion of COL17 disturb the supply of hemidesmosomal COL17 and impair hemidesmosome formation. Eventually, intra-lamina lucida separation is caused by mechanical stress, complement activation, and/or inflammatory cell infiltration. This is mainly observed in the skin; therefore, the blisters predominantly occur in the skin (left panel). In the oral mucosa, autoantibodies targeting the C-terminus of COL17 enhance COL17 depletion induced by autoantibodies targeting COL17-NC16A. The blister formation in oral mucosa may be a result of the enhancement of COL17 depletion induced by autoantibodies targeting the C-terminus of COL17 in BP patients (right panel). **(B)** The predominant oral mucosal blistering in MMP. The direct binding of COL17 to COL4 is disrupted by IgG against the C-terminus in the oral mucosa. Autoantibodies in MMP targeting the C-terminus of COL17 inhibit the protein–protein interaction in the oral mucosa and reduce hemidesmosomal adhesion without the internalization of COL17.

Recently, we gained new insight into the blister mechanism of oral lesions (81). We showed that COL17 expression is approximately 30–50% higher in mucosal keratinocytes than in skin keratinocytes, as confirmed by qPCR and immunoblotting analysis. This higher COL17 expression in mucosal keratinocytes is associated with stronger cell adhesion in such keratinocytes. The cell adhesion strength was found to be 50% higher for mucosal keratinocytes than skin keratinocytes. Furthermore, anti-COL17-NC16A antibodies induce significantly greater COL17 depletion in skin keratinocytes than in mucosal keratinocytes. This indicates that the higher expression of COL17 in mucosa may compensate for the COL17 depletion induced by pemphigoid IgG. In other words, the predominant skin blistering may relate to the residual amount of COL17 after BP-IgG induces COL17 depletion. This is similar to the blistering mechanism in pemphigus, in which desmoglein 1 and 3 compensate for each other in the oral mucosa and the skin. Potential blister formation in BP is initiated by the binding of autoantibodies to COL17, which leads to the internalization and depletion of COL17 from the plasma membrane. The depletion of COL17 may impair hemidesmosome formation and weaken the strength of adhesion to the basement membrane. Finally, separations may be caused by mechanical stress or inflammation induced via the Fc fragment of the pathogenic IgG. The lower frequency of oral lesions in BP may be attributed to the high expression level of COL17 in oral mucosa (Figure 2A). Additionally, we showed that IgG against the C-terminus of COL17 may have pathogenicity. The pathogenicity of IgG against regions outside the NC16A domain remains controversial *in vivo* and *in vitro* (74, 82, 83). In our study, however, COL17 depletion was significantly enhanced by stimulation with a combination of IgGs against the NC16A domain and the C-terminus (Figure 2A). This evidence suggests that BP patients with IgG targeting not only the NC16A domain but also the C-terminus may show blisters in the skin and the mucosa. To support this, several studies demonstrated an association between autoantibodies to the C-terminus and mucosal lesions in BP (64, 84). Autoantibodies targeting the C-terminus are potentially pathogenic in certain cases of BP.

### MMP-Specific Blister Mechanism Without Inflammation

Histologically, MMP patients have less severe inflammatory findings than BP patients do. The blistering mechanism of MMP may differ from that of BP. We recently found direct binding between collagen IV (COL4) and COL17 in skin and oral keratinocytes (24). Interestingly, this COL4–COL17 binding is disrupted by IgG against the C-terminus in oral keratinocytes. Furthermore, several MMP IgGs that target the C-terminus of COL17 were found to inhibit COL4–COL17 binding and to result in the reduction of hemidesmosomal adhesion (Figure 2B). That is, MMP-IgGs may directly disrupt COL4-COL17 binding and result in separation at the BMZ without inflammation.

As for the potential blistering mechanism of laminin 332-type MMP, Fc-dependent, and complement-dependent mechanisms have been revealed by using laminin 332-type mouse models. However, laminin 332 interacts with other BMZ molecules, including COL17. Given our latest concept of MMP-specific blister formation, anti-laminin 332 antibodies may disrupt the molecular interactions of laminin 332, resulting in the predominance of mucosal blister formation in laminin 332-type MMP.

## Conclusion

As highlighted in this review, we propose disease-specific diagnostic strategies for MMP. The pathogenesis of COL17-type MMP is distinct from that of BP and is more closely related to less inflammatory blister mechanisms due to the inhibition of COL4–COL17 binding or COL17 depletion.

## Ethics Statement

The studies were conducted in accordance with the Helsinki Guidelines and were approved by the Ethics Committee of Hokkaido University.

## Author Contributions

All authors listed have made a substantial, direct and intellectual contribution to the work, and approved it for publication.

### Conflict of Interest Statement

The authors declare that the research was conducted in the absence of any commercial or financial relationships that could be construed as a potential conflict of interest.

## Abbreviations

MMP: mucous membrane pemphigoid
BMZ: basement membrane zone
COL17: collagen XVII
COL7: collagen VII
BP: bullous pemphigoid
DIF: direct immunofluorescence
IIF: indirect immunofluorescence
ssIIF: 1 M NaCl-split skin IIF
ELISA: enzyme-linked immunosorbent assays
NC: non-collagenous
CLEIA: chemiluminescence enzyme immunoassay
DPP-4i: dipeptidyl peptidase-4 inhibitor
COL4: collagen IV.
